# Implant surgery in healthy compromised patients-review of literature

**Published:** 2014

**Authors:** IM Gheorghiu, IM Stoian

**Affiliations:** * Department of Operative Dentistry, Faculty of Dental Medicine, “Carol Davila” University of Medicine and Pharmacy, Bucharest; ** Department of Biochemistry, Faculty of Medicine, “Carol Davila” University of Medicine and Pharmacy, Bucharest

**Keywords:** dental implant, healthy compromised patients, limitations, complications

## Abstract

Systemic diseases are of major importance in terms of prosthetic restorations supported by dental implants in healthy compromised patients. Each treatment stage from conception of the treatment plan to the long-term monitoring is under the necessity of the interdisciplinary approach to the underlying disease.

**Abbreviations:** healthy compromised patients = HCP

**Introduction**

A healthy compromised patient can benefit from oral implant rehabilitation, which will significantly increase the patient quality of life and sometimes will even improve its medical condition.

**Dental implant therapy in healthy compromised patients**

What should be kept in mind when dealing with healthy compromised patients is that there is a downward change of the reserves and general reactivity of the body. These predispose to various complications during surgery and surgery-immediate or on long term. Hence, the necessity of precautions that must be applied to such patients would arise during the surgical act, but to the same extent in the prosthetic phase or the in monitoring period [**1**,**2**].

Systemic disorders affect the implant restoration therapy for the following reasons:

1. Inter-relationships between body and oral cavity. By the time the patient presented to treatment by implants, the systemic disease would already have had repercussions in the oral cavity. It is known that many general maladies stand out particularly in the oral cavity, for example, patients suffering from diabetes have a periodontal disease with an aggressive evolution compared to non-diabetic patients [**3**]. 

2. Medications the patient receives for his systemic chronic diseases, always have a number of side effects [**4**]. They have consequences both to the mouth, and to the whole body: abnormal bleeding (non-steroidal drugs anti-inflammatory), altered hematopoiesis (barbiturates), decreased stress tolerance (beta-blockers), gingival hyperplasia (nifedipine), respiratory depression (narcotics), altered host resistance (insulin, antibiotics), xerostomia (many drugs). These are factors that limit and condition a number of therapeutic measures, especially in the surgical stage of dental implant therapy [**5**,**6**]. 

3. The systemic diseases can lead to accidents and intraoperative complications. For example, a patient with heart disease can go into cardiac depression during implant surgery while endangering the vital functions.

4. The dental implant therapy in patients with systemic disorders should be designed while taking into account long-term complications that may arise; these kind of complications are more frequent and severe than in a healthy patient. They can completely undermine the surgery. For example, in the case of a patient with osteoporosis, a bone resorption, which is characteristic for the general disease, can increase the time necessary for osseointegration; also, there is a need for a specific manner for prosthetic loading, designed to stimulate bone healing [**7**]. 

The most common types of systemic diseases we are facing are the following:

-cardiovascular disease: hypertension, atherosclerosis, valvular heart diseases, angina pectoris;

-endocrine disorders: diabetes, thyroid disease, adrenal gland disorders;

- hematologic disorders: anemia, leukemia;

- lung: emphysema, chronic bronchitis;

-liver disease: cirrhosis;

-disease of bone: osteoporosis, hyperparathyroidism;

Elder patients are also classified as healthy compromised patients because they present multiple associated diseases in addition to physiological changes due to age involution [**8**,**9**].

Patients who smoke show changed general and local medical conditions, so, possible complications and special preventive measures will be taken into account.

The treatment plan design and the accomplishment of surgical and prosthetic phases in HCP are a consequence of the full evaluation of general health and associated diseases. 

One of the most important aspects is antibiotic prophylaxis in the treatment of HCP with dental implants. It is mandatory in patients with myocardial infarction, those with an increased risk of bacterial endocarditis, those with diabetes, or those with an immune suppression by corticosteroids or radiation therapy [**10**]. In addition, the antibiotic prophylaxis is recommended in patients with anemia or liver disorders.

In HCP, the whole treatment should also be related to the clinical laboratory tests, they are necessary in such cases. The most common clinical laboratory parameters investigated are the following: complete blood cell count (CBCC), bleeding tests, serum glucose, serum calcium, creatinine, bilirubin or other indicators [**11**]. 

Stress reduction protocol is the key factor in treatment planning and surgery procedures in healthy compromised patients:

1. Patients with cardiovascular diseases. In the case of patients with hypertension, the hypotensive medication that they receive has the side effect of xerostomia, consecutive fungal infections of the mouth (candidiasis). Meanwhile, calcium blockers also used as hypotensive medication, produce gingival hyperplasia, erythema and ulcers, both in the natural dentition and around the implant. Interventions involving patients with cardiovascular diseases should be the following: as shorter treatment sessions as possible and a cautious use of vasoconstrictor substances in anesthesia, due to the risk of intraoperative episode of angina pectoris.

Consecutive transient bacteremia is directly proportional to the amount of oral tissue trauma. In patients with heart diseases, it can cause bacterial endocarditis with very serious consequences. Therefore, surgery should be as less aggressive as possible. Antibiotic prophylaxis of bacterial endocarditis is necessary in patients with high risk: previous endocarditis, prosthetic heart valve, rheumatic valvular defect, congenital heart disease [**12**].

The patients who take anticoagulants demand surgery procedures, which are less invasive, for example the flapless insertion of the dental implant. The bleeding is reduced to a minimum and the associated risks are reduced. The actual attitude towards these patients is to keep the anticoagulant medication because the risks of a stroke or myocardial infarction are more important than the intraoperative bleeding. Prior to surgery, in the same day, the prothrombin time test should be done [**13**]. 

2. Patients with endocrine disorders. Diabetes is one of the most common diseases we generally face in dental practice. The effects of this disease in the oral cavity are the following: aggressive periodontal disease, alveolar bone loss, increased risk of inflammation and infection of gums, dry mouth, candidiasis [**14**]. In terms of surgical approach, the patient with diabetes should be applied an operator stress reduction protocol to prevent intraoperative hyperglycemia crisis. This protocol includes the following: control diet and hypoglycemic medication, special aseptic techniques and rigorous antibiotic prophylaxis [**15**,**16**]. 

It is important to know that the intervention of inserting dental implants may trigger a congestive heart failure, or on the contrary may initiate a cardio-respiratory depression in patients who suffer from thyroid disorders (hyper- and hypothyroidism). Patients with chronic adrenal insufficiency (Addison diabetes) may be at the same risk during surgical procedures. The hypofunction of the adrenal glands (Cushing's syndrome) has consequences on both the condition of the patient (hypertension) and also the healing is delayed, with the risk of secondary infection. Long-term corticosteroid therapy affects the dental implant. Immediate postoperative effects are beneficial by reducing inflammation and pain, but corticosteroids delay the healing, decrease the blood leukocyte count and decrease the patient's ability of antibacterial defense [**17**].

3. Blood disorders are some of the most critical diseases with echo in oral implantology. Anemia causes complications both on short term and long term: delayed healing, decreased bone density, increased healing time. The intraoperative bleeding in such patients is high; the consequences for the patient are the possibility of postoperative edema and increased discomfort. It is associated with a high risk of secondary infections. Long-term implant survival is low due to frequent chronic infections. Leukocyte disorders induce multiple complications that can compromise the success of the implant. The most common of these is infection (it can occur during any stage of treatment). Just like in patients with anemia, intraoperative bleeding is high, and the risk of postoperative edema and secondary infection are increased.

4. Patients suffering from chronic pulmonary affections can receive an implant, but the medication for their specific pathology is with corticosteroids, producing a suppression of the body. As such, its response to surgical stress is altered. The possible complications have already been mentioned.

5. Liver diseases interfere with surgical procedures by affecting the process of hemostasis. Intraoperative bleeding is increased in such patients, the consequences being listed above. Low capacity to metabolize medicinal substances can lead to respiratory depression.

6. Skeletal disorders are particularly related to changes of bone resorption. Osteoporosis is present in the jawbone where the implant will be inserted, having the same features as the other bones of the body: thinning of cortical bone, decrease of trabecular pattern and demineralization. Osteoporosis itself is not a systemic disease that contraindicates the dental implant, but it affects every stage of the treatment in a particular way: the plan of the treatment (choice of type of implants that provide a larger contact area with the bone), surgical approach as conservative and less traumatic as possible [**18**]. What is specific for this disease is its influence on the prosthetic phase: the need for a progressive load of the implant in order to stimulate the increasing bone density [**19**].

Another important aspect related to osteoporosis is the medication used for this disorder: bisphosphonates (BPs) are an important group of drugs. Osteonecrosis of the jaw is a complication observed in patients who use oral or intravenous bisphosphonates. It is called bisphosphonate-related osteonecrosis of the jaw (BRONJ). It is important to inform all the patients undergoing bisphosphonate therapy about the possible risks of development of osteonecrosis [**20**,**21**]. All the patients undergoing implant placement should be questioned about the bisphosphonate therapy including the drug taken, the dosage, and length of treatment prior to surgery because all these factors influence the protocol of treatment [**22**,**23**]. 

Hyperparathyroidism affects the jaws as osteoporosis does: loss of lamina dura, altered trabecular bone pattern. The effects on dental implants are the same with the ones for patients with osteoporosis.

7. Patients over 60 years old have a physiological adaptability to diminished stress, which is why they are treated similar to patients with mild medical general conditions. It requires a high level of caution intra-and post-operatively. If there are systemic disorders, they are taken into account in determining the therapeutic approach. Medications that seniors routinely take, interfere with the oral cavity status by the side effects [**24**,**25**]. 

8. Smoking is a vicious habit with systemic and local complications. Special measures have to be taken, mostly related to a delayed healing of the soft tissue, the decreased resistance to inflammation of the gums and tissues around the implant and decreased resistance to infection after any oral surgery. The systemic diseases affect the patients' pre-operative status and also the surgical and prosthetic phases [**26**,**27**]. Depending on the type of systemic pathology, the special precautions needed to be taken in HCP are the following: 

- For the prevention of intraoperative complications such as angina attack or cardiovascular-respiratory depression: the operative protocol as less stressful as possible, avoiding major interventions with a long duration of time; first aid kit ready to hand [**28**]; 

- To achieve hemostasis: making special types of sutures or by using collagen sponges;

- To decrease the risk of secondary infection: antibiotic prophylaxis and antibiotics therapy [**29**];

- For proper implant osseointegration: implant systems and specific ways of achieving their prosthetic loading.

**Conclusions**

It is mandatory to know the implications of the systemic diseases or those produced by current medications in the oral cavity, in order to prevent failures in HCP patients who receive dental implant therapy.

Given the altered biological constants of the patient, the medical team must be able to solve any complication that occurs and should also be prepared with more treatment options for that situation.
